# Emerging Trends in Alternative and Sustainable Crop Production

**DOI:** 10.3390/plants15020306

**Published:** 2026-01-20

**Authors:** Dimitrios Bilalis, Ioanna Kakabouki, Ioannis Roussis

**Affiliations:** Laboratory of Agronomy, Department of Crop Science, Agricultural University of Athens, 11855 Athens, Greece; bilalis@aua.gr

The last few decades have seen the evolution of crop systems management studies from the traditional production-based agronomy domain, not only as a way of addressing the challenges of contemporary agriculture but also as a result of the complexity of these challenges [1]. Modern crop systems management studies take an integrated approach that includes aspects such as agronomic, ecological, environmental, social, and economic aspects of crop systems management, with the development of new technologies as a key aspect of this approach [2,3,4,5]. Understanding the biological foundation of sustainability is key to this approach, which is centered on meeting the current agricultural needs without jeopardizing the ability of future generations to meet their needs [6].

In this scenario, the current Special Issue (SI) seeks to assess the sustainability of cropping systems by dealing with major global questions as well as recent developments in environmentally sound and alternative agricultural practices/technologies. In particular, the SI considers various themes such as the use of waste resources, adaptation to or the mitigation of the effects of climate change, management of natural resources, enhancement of soil health by carbon sequestration, efficient management of water/nutrients, reduction in pollution, and conservation of biodiversity. The SI includes five papers (three research articles, one review article, and one perspective paper) that together establish the trends and shifts towards the sustainability of the global cropping system.

Water deficiency and poor water use efficiency greatly hinder wheat yields in dryland areas [7]. Tillage systems and straw management systems had been investigated by Dong et al. [8] for dry matter production, grain yield, and water use efficiency in dryland winter wheat and summer soybean double cropping in a field experiment established in 2009 in China. Two systems of tillage (plowing and rotary tillage) were used together or without straw mulching. The results revealed that the effect of straw mulching significantly promoted wheat yield and components independent of tillage systems, but their interaction significantly affected most characteristics. Among these systems, rotary tillage and straw mulching (RTSM) performed best by increasing the wheat yield due to increments in spike number, number of grains per spike, 1000-grain weight, and harvest index. The cause for such higher yields could be attributed to increments in both pre-and post-anthesis dry matter, an increase in dry matter translocation to the grain, an increase in pre-sowing soil water storage capacity, and improvement in water use efficiency. Additionally, an evaluation using Technique for Order Preference by Similarity to Ideal Solution confirmed that the reason for the better performance in RTSM is largely due to increments in biomass and water productivity due to straw management practices. This clearly reveals that managing dryland wheat and soybean double cropping through rotary tillage and straw mulching is effective and efficient for boosting wheat yields.

Use of microbial consortia-based biofertilizers is a promising approach for realizing sustainable agriculture in the face of challenges to ecological and agricultural systems worldwide [9]. The agroecological and economic effectiveness of these biofertilizers was investigated by Nygymetova et al. [10] for a technology transfer study in China and Kazakhstan as a model for countries of different levels of scientific and technical capacity. In China, effective adoption is ensured by government commitments to development research and a well-developed scientific base. In Kazakhstan, factors such as the absence of financial support and an inadequate technology transfer system remain existing obstacles to the successful adaptation of biofertilizer technology despite a positive agroecological environment and increasing interest from farmers. This study illustrates a specific need for the right adaptation of innovative advances from one country to another, with due attention to a country’s specific socioeconomic settings and enhanced intergovernmental collaboration to realize sustained productivity of agricultural systems and food security in a nation, whatever its level of economic development.

Root knot nematodes (RKN) are very harmful to crops, but little is known about hemp susceptibility and the post-control methods. Ntinokas et al. [11] investigated the pathogenicity of the following five species of RKNs (*Meloidogyne javanica*, *M. incognita*, *M. arenaria*, *M. hapla*, and *M. luci*) for nine industrial hemp varieties and the nematicidal activity of aqueous and ethanolic hemp seed extracts against *M. javanica* juveniles. All the nematodes were pathogenic, with *M. javanica* and *M. luci* having the lowest shoot weight and *M. incognita* with the most severe root damage. Susceptible varieties were USO 31, Fedora 17, Ferimon 12, and Zenit, with the lowest nematode infestation for Futura 75. Both extracts paralyzed the highly concentrated juveniles, but the ethanolic extraction was active from >62.5 ppm and resulted in up to 76% inhibition of egg hatchability, while the aqueous extraction was less potent. From the experiment, hemp is susceptible to *M. javanica* infection, and the ethanolic hemp seed extraction is an active biocontrol method against *M. javanica* infection in hemp plants.

According to recent research studies, it was found that the tetraploid rice shows an enhanced grain-filling rate, especially in new hybrids, leading to higher yields compared to previous levels. Based on this, Xiao et al. [12] examined the influence of the application of nitrogen fertilizer and planting density on growth, development, yield, and nitrogen use in tetraploid rice (T7, an *indica–japonica* conventional allotetraploid) and diploid rice (FLY4, a two-line super hybrid as control). The work demonstrated that the maximum grain-filling rate for T7 was 77.8%, which marked a substantial increase compared to the earlier tetraploid varieties. However, the grain yield of T7 remained lower compared to that of the diploid rice FLY4, mainly because of the low grain-filling efficiency, number of spikelets per panicle, panicle per square meter, and harvest index. The differences in the application of nitrogen fertilizer and the level of planting density caused negligible differences in the grain yield of the two varieties. Increased planting density caused an elevation in the leaf area index and biomass, along with a decrement in the harvest index, showing that the nitrogen use efficiency in FLY4 was substantially higher compared to T7, mainly due to the greater use of the former in the straw component. Greater application of the fertilizer caused a decline in the use efficiency of the fertilizer, mainly because it increased fertilizer use without resulting in a corresponding increase in the grain yield. Based on the observations, it appears that further improvement in the yield of tetraploid rice can be achieved through the improvement of the grain-filling rate, panicle number, and the number of spikelets per panicle.

The debate about sustainable agriculture towards a more sustainable agriculture system has begun to disqualify modern, “industrial” agriculture for its role in harming the natural environment and its biodiversity, even if it provides more than 90% of the food for the worldwide population [13,14]. Lenné and Wood [15], in a critique of alternative approaches to sustainable agriculture, hold up for scrutiny not only “agroecology” but, within the category of agroecologies, the “Vision for Adapted Crops and Soils” (VACS) initiative and its application to the developing nations of the Global South. In addition, this perspective paper stresses the availability of diversification methods in modern agriculture and the inefficacy of replacing chemical fertilizer with organic fertilizer in degraded soils. In the case of VACS, they question its perspective on the significance of local and indigenous crops in the face of the significance of non-indigenous crops to food security, particularly in Africa. Finally, the authors ultimately argue for the integration of every strategy currently available for agriculture, with the goal of achieving sustainable food production and agriculture.

In summary, these papers illustrate that it is imperative to cover agronomic, ecological, and socio-economic aspects in ensuring that sustainable crop production is enhanced. This requires context-driven innovation and management as well as strategic use of practices that address contemporary agricultural and future resource conservation needs.

## References

[1] Dixon J., Li L., Amede T. (2023). A Century of Farming Systems. Part 1: Concepts and Evolution. Farming Syst..

[2] Dessart F.J., Barreiro-Hurlé J., van Bavel R. (2019). Behavioural Factors Affecting the Adoption of Sustainable Farming Practices: A Policy-Oriented Review. Eur. Rev. Agric. Econ..

[3] Medhekar A., Machado C.F., Davim J.P. (2024). Circular Economy in Agriculture and Sustainable Development. Circular Economy and Manufacturing.

[4] Boros A., Szólik E., Desalegn G., Tőzsér D. (2025). A Systematic Review of Opportunities and Limitations of Innovative Practices in Sustainable Agriculture. Agronomy.

[5] Terán-Samaniego K., Robles-Parra J.M., Vargas-Arispuro I., Martínez-Téllez M.Á., Garza-Lagler M.C., Félix-Gurrlola D., Maycotte-de la Peña M.L., Tafolla-Arellano J.C., García-Figueroa J.A., Espinoza-López P.C. (2025). Agroecology and Sustainable Agriculture: Conceptual Challenges and Opportunities—A Systematic Literature Review. Sustainability.

[6] Pradhan N.C., Gavhane K.P., Bhalekar D.G., Dharmender, Kiran P.R., Sarkar T., Smaoui S. (2026). Sustainable Agriculture Fundamentals. Health, Nutrition and Sustainability.

[7] Zhang D., Yao P., Na Z., Cao W., Zhang S., Li Y., Gao Y. (2016). Soil Water Balance and Water Use Efficiency of Dryland Wheat in Different Precipitation Years in Response to Green Manure Approach. Sci. Rep..

[8] Dong S., Huang M., Zhang J., Zhou Q., Hu C., Liu A., Wang H., Fu G., Wu J., Li Y. (2025). Long-Term Rotary Tillage and Straw Mulching Enhance Dry Matter Production, Yield, and Water Use Efficiency of Wheat in a Rain-Fed Wheat-Soybean Double Cropping System. Plants.

[9] Nunes P.S.O., Lacerda-Junior G.V., Mascarin G.M., Guimarães R.A., Medeiros F.H.V., Arthurs S., Bettiol W. (2024). Microbial Consortium of Biological Products: Do They Have a Future?. Biol. Control.

[10] Nygymetova A.M., Sadvakasova A.K., Zaletova D.E., Kossalbayev B.D., Bauenova M.O., Wang J., Huang Z., Sarsekeyeva F.K., Kirbayeva D.K., Allakhverdiev S.I. (2025). Development and Transfer of Microbial Agrobiotechnologies in Contrasting Agrosystems: Experience of Kazakhstan and China. Plants.

[11] Ntinokas D., Roussis I., Mavroeidis A., Stavropoulos P., Folina A., Kakabouki I., Tzortzakakis E.A., Bilalis D., Giannakou I.O. (2025). Virulence of Five Root-Knot Nematodes (*Meloidogyne* spp.) on Nine Industrial Hemp (*Cannabis sativa* L.) Varieties and Nematicidal Potential of Hemp Seed Extracts Against *Meloidogyne javanica*. Plants.

[12] Xiao J., Xiong Z., Huang J., Zhang Z., Cai D., Xiong D., Cui K., Peng S., Huang J. (2024). Differences in Grain Yield and Nitrogen Uptake between Tetraploid and Diploid Rice: The Physiological Mechanisms under Field Conditions. Plants.

[13] McIntyre B.D., Herren H.R., Wakhungu J. (2009). Agriculture at the Crossroads. Report of the International Assessment of Agricultural Knowledge, Science and Technology.

[14] Smith V.H., Goodwin B.K. (2024). World Agricultural Crop Production over the Past Six Decades: A Miracle of Science, Technology, Innovation, and Entrepreneurship.

[15] Lenné J., Wood D. (2025). The Promotion of Alternative Crop Production Paradigms Should Be Founded on Proven Science-Based Approaches. Plants.

